# Tissue source determines the differentiation potentials of mesenchymal stem cells: a comparative study of human mesenchymal stem cells from bone marrow and adipose tissue

**DOI:** 10.1186/s13287-017-0716-x

**Published:** 2017-12-06

**Authors:** Liangliang Xu, Yamei Liu, Yuxin Sun, Bin Wang, Yunpu Xiong, Weiping Lin, Qiushi Wei, Haibin Wang, Wei He, Bin Wang, Gang Li

**Affiliations:** 1 Key Laboratory of Orthopaedics & Traumatology, The First Affiliated Hospital of Guangzhou University of Chinese Medicine, The First Clinical Medical College, Guangzhou University of Chinese Medicine, Guangzhou, China; 2 Departments of Diagnostics of Traditional Chinese Medicine, Guangzhou University of Traditional Chinese Medicine, Guangzhou, Guangdong 510006 People’s Republic of China; 3 Department of Orthopaedics & Traumatology, Faculty of Medicine, The Chinese University of Hong Kong, Prince of Wales Hospital, Shatin, Hong Kong, Special Administrative Region of China; 4 Key Laboratory for Regenerative Medicine, Ministry of Education, School of Biomedical Sciences, Faculty of Medicine, The Chinese University of Hong Kong, Hong Kong, SAR China; 5 Stem Cells and Regenerative Medicine Laboratory, Lui Che Woo Institute of Innovative Medicine, Li Ka Shing Institute of Health Sciences, The Chinese University of Hong Kong, Prince of Wales Hospital, Shatin, Hong Kong, Special Administrative Region of China; 6 The CUHK-ACC Space Medicine Centre on Health Maintenance of Musculoskeletal System, The Chinese University of Hong Kong Shenzhen Research Institute, Shenzhen, People’s Republic of China; 7 Department of Traumatology, The Third Affiliated Hospital of Guangzhou University of Traditional Chinese Medicine, Guangzhou, Guangdong 510240 People’s Republic of China; 8 Room 904, 9/F, Li Ka Shing Institute of Health Institute, Prince of Wales Hospital, The Chinese University of Hong Kong, Shatin, Hong Kong, Special Administrative Region of China; 9 Department of Traumatology, The First Affiliated Hospital of Guangzhou University of Traditional Chinese Medicine, Guangzhou, China

**Keywords:** Mesenchymal stem cells, Epigenetic regulation, Bone marrow-derived MSCs, Adipose tissue-derived MSCs

## Abstract

**Background:**

Mesenchymal stem cells (MSCs) possess intrinsic regeneration capacity as part of the repair process in response to injury, such as fracture or other tissue injury. Bone marrow and adipose tissue are the major sources of MSCs. However, which cell type is more effective and suitable for cell therapy remains to be answered. The intrinsic molecular mechanism supporting the assertion has also been lacking.

**Methods:**

Human bone marrow-derived MSCs (BMSCs) and adipose tissue-derived MSCs (ATSCs) were isolated from bone marrow and adipose tissue obtained after total hip arthroplasty. ATSCs and BMSCs were incubated in standard growth medium. Trilineage differentiation including osteogenesis, adipogenesis, and chondrogenesis was performed by addition of relevant induction mediums. The expression levels of trilineage differentiation marker genes were evaluated by quantitative RT-PCR. The methylation status of CpG sites of Runx2, PPARγ, and Sox9 promoters were checked by bisulfite sequencing. In addition, ectopic bone formation and calvarial bone critical defect models were used to evaluate the bone regeneration ability of ATSCs and BMSCs in vivo.

**Results:**

The results showed that BMSCs possessed stronger osteogenic and lower adipogenic differentiation potentials compared to ATSCs. There was no significant difference in the chondrogenic differentiation potential. The CpG sites of Runx2 promoter in BMSCs were hypomethylated, while in ATSCs they were hypermethylated. The CpG sites of PPARγ promoter in ATSCs were hypomethylated, while in BMSCs they were hypermethylated. The methylation status of Sox9 promoter in BMSCs was only slightly lower than that in ATSCs.

**Conclusions:**

The epigenetic memory obtained from either bone marrow or adipose tissue favored MSC differentiation along an osteoblastic or adipocytic lineage. The methylation status of the main transcription factors controlling MSC fate contributes to the differential differentiation capacities of different source-derived MSCs.

**Electronic supplementary material:**

The online version of this article (doi:10.1186/s13287-017-0716-x) contains supplementary material, which is available to authorized users.

## Background

Mesenchymal stem cells (MSCs) possess intrinsic regeneration capacity as part of the repair process in response to injury, such as fracture or other tissue injury. Several characteristics of MSCs, such as the potential to differentiate into multiple lineages and the ability to be easily expanded ex vivo while retaining their original lineage differentiation commitment, make these cells very promising targets for therapeutic use in regenerative medicine and tissue engineering [1]. However, low cell survival rate and differentiation capacity in vivo after MSC transplantation have significantly reduced the effectiveness of stem cell therapy [2–5]. Over the past decade, MSCs have been isolated from the umbilical cord, umbilical cord blood, bone marrow, adipose tissue, and many other adult tissues. To date, bone marrow-derived MSCs (BMSCs) and adipose tissue-derived MSCs (ATSCs) are still the main source of MSCs, especially in autologous cell-based therapies, due to ease of harvest and potential autologous application [6]. An important question,  which type of cell is more effective and suitable for cell therapy remains unknown. Many studies have shown that BMSCs and ATSCs share similar features, including the morphology and cell surface markers, but significant biologic differences have been found concerning their proliferation and differentiation capacities [7–10]. Conflicting results have been reported; some studies indicate that the clinical application potential of ATSCs is more effective than or as effective as that of BMSCs, while other studies conclude that BMSCs are superior to ATSCs [11–13]. In addition, significant differences in the cytokine secretome and chemokine receptor expression between ATSCs and BMSCs have also been reported [14, 15]. Despite different gene expression and differentiation capacities have been observed among ATSCs and BMSCs, the underlying mechanisms regarding epigenetic regulation are yet to be investigated.

Interestingly, recent studies from both our group and others have demonstrated that epigenetic regulation is an important factor to control MSC differentiation and proliferation [16]. Up to now, DNA methylation and histone modifications are the most important epigenetic regulations which possess the power to control the differentiation or maintain the self-renewal of MSCs [17]. Changes in the methylation states of the CpG islands in the promoter regions or the first exon are known to be inversely responsible for expression of the corresponding genes. The bivalent loci in MSCs are often low in DNA methylation and can be further methylated or activated, which are distinct from those in the embryonic stem cells and differentiated cells [18]. Targeted DNA methylation within the Trip10 promoter has been shown to accelerate the MSCs to neuron or osteocyte differentiation [19].

In the present study, we determined the effect of epigenic regulation of MSC fate. The results showed that epigenetic memory obtained from either bone marrow or adipose tissue favored their differentiation along an osteoblastic or adipocytic lineage. The CpG sites of Runx2 promoter in BMSCs were hypomethylated, while in ATSCs they were hypermethylated. The CpG sites of PPARγ promoter in ATSCs were hypomethylated, while in BMSCs they were hypermethylated. The methylation status of Sox9 promoter in BMSCs was only slightly lower than that in ATSCs. We concluded that the methylation status of the main transcription factors controlling MSC fate contributed to the differential differentiation capacities of different source-derived MSCs.

## Methods

### Isolation and culture of human BMSCs and ATSCs

ATSCs and BMSCs were prepared as described previously [20, 21]. Briefly, the BMSCs were fractionated on a Ficoll density gradient (Ficoll-Paque™-PLUS; Amersham Pharmacia, Sweden) and the MSC-enriched fraction was washed, seeded in culture flasks, and maintained at 37 °C in a humidified atmosphere. The adipose tissue was washed extensively with equal volumes of phosphate-buffered saline (PBS), and the extracellular matrix was digested with 0.075% collagenase (type I; Sigma-Aldrich, St Louis, MO, USA) at 37 °C for 30 min. With α-MEM containing 10% FBS and antibiotics (100 U/ml penicillin G and 100 μg/ml streptomycin), the sample was centrifuged at 1200 × *g* for 10 min. The cell pellet was resuspended in 160 mM NH_4_Cl and incubated at room temperature for 10 min. After removing cellular remains through a 100-μm Nylon mesh (Cell Strainer; Becton Dickinson and Company, Franklin Lakes, NJ, USA), the cells were incubated in the culture medium. The adhered ATSCs were cultured for about 2 weeks, and nearly all cells transformed into fibroblast-like cells, which are morphologically similar to BMSCs.

### Phenotypic characterization of hMSCs

After reaching 80% confluence, the cells were rinsed twice with PBS and treated with 0.05% trypsin–EDTA for 2 min. Serum-containing medium was then immediately added to the culture to end trypsinization. The fluid was then collected and centrifuged (800 × *g* for 5 min). After discarding the supernatant, the precipitate was resuspended in staining buffer and incubated with fluorochrome-conjugated primary antibodies against CD34, CD44, CD45, CD73, CD90, and CD105 or corresponding isotype control (BD Biosciences, USA) at 4 °C for 30 min. The stained cells were immediately detected using flow cytometry (BD Biosciences, USA).

### Osteogenic differentiation

MSCs were plated at 4 × 10^3^ cells/cm^2^ in a 12-well plate and cultured in the basal medium until the cells reached confluence. The cells were then incubated in osteogenic induction medium (OIM), which is basal medium supplemented with 1 nM dexamethasone, 50 μM ascorbic acid, and 20 mM β-glycerolphosphate (all from Sigma-Aldrich), at 37 °C, 5% CO_2_ as described previously [20, 21]. At day 14, the mineralization of MSCs was assessed by Alizarin Red S staining. Briefly, to evaluate the mineralized nodule formation in vitro, the cell/matrix layer was washed with PBS, fixed with 70% ethanol for 10 min, and stained with 0.5% Alizarin Red S (pH 4.1; Sigma, St Louis, MO, USA) for 5 min.

### Adipogenic differentiation

MSCs were plated at 4 × 10^3^ cells/cm^2^ in a six-well culture plate and cultured until the cells reached confluence. The medium was then replaced with adipogenic induction medium (AIM), which is basal medium supplemented with 500 nM dexamethasone, 0.5 mM isobutylmethylxanthine, 50 mM indomethacin, and 10 mg/ml of insulin (all from Sigma-Aldrich). The cells were cultured for another 21 days, and then the cells were fixed with 70% ethanol for 10 min and stained with 0.3% fresh Oil Red O solution (Sigma-Aldrich) for 10 min. The wells were rinsed three times with distilled water and viewed using a LEICA Q500MC microscope (Leica Cambridge Ltd).

### Chondrogenic differentiation

For chondrogenic differentiation, a micromass culture system was used. MSCs (in 5 μl) at a centration of 1.6 × 10^7^ cells/ml were dropped in the centers of 24-well plates. The plates were placed in incubator at 37 °C, 5% CO_2_ without culture medium for 2 hours. These cells were then cultured in chondrogenic induction medium (CIM), which is basal medium supplemented with 10 ng/ml transforming growth factor-β3 (R&D Systems), 500 ng/ml bone morphogenetic protein-2 (R&D Systems), 10^–7^ M dexamethasone, 50 mg/ml ascorbate-2-phosphate, 40 mg/ml proline, 100 mg/ml pyruvate (all from Sigma-Aldrich), and 1:100 diluted ITS + Premix (6.25 mg/ml insulin, 6.25 mg/ml transferrin, 6.25 mg/ml selenous acid, 1.25 mg/ml bovine serum albumin, and 5.35 mg/ml linoleic acid) (Becton Dickinson). The chondrogenic medium was changed every 3 days.

### Quantitative real-time RT-PCR

The cells were harvested and homogenized for RNA extraction with the RNeasy mini kit (Qiagen, Hilden, Germany). The mRNA was reverse-transcribed to cDNA by the PrimeScript First Strand cDNA Synthesis Kit (TaKaRa). Then 5 μl of total cDNA from each sample was amplified in a final volume of 25 μl of reaction mixture containing Platinum SYBR Green, qPCR SuperMix-UDG ready-to-use reaction cocktail, and specific primers using the ABI StepOne Plus system (all from Applied Biosystems, CA, USA). The expression level of the target gene was normalized to that of the β-actin gene, which was shown to be stable in this study. Relative gene expression was calculated with the 2^–△CT^ formula. The sequences of the primers were presented in Additional file 1: Table S1.

### DNA isolation and bisulfite treatment

Genomic DNA was isolated from MSCs using the PureLink® Genomic DNA isolation kit following the manufacturer’s instructions (Invitrogen). Bisulfite modification was done as described previously [22]. Briefly, about 2 μg of genomic DNA was denatured by NaOH (final concentration 0.2 mol/L) for 10 min at 37 °C. Hydroquinone and sodium hydroxide were added, and samples were incubated at 50 °C for 16 hours. Modified DNA was purified using the Wizard DNA Clean-Up System following the manufacturer’s instructions (Promega) and eluted into 50 μl of water. DNA was treated with NaOH (final concentration 0.3 mol/l) for 5 min at room temperature, ethanol precipitated, and resuspended in 20 μl of water. Modified DNA was used immediately or stored at –20 °C.

### Bisulfite sequencing

Bisulfite-modified genomic DNA was amplified by PCR. All PCRs were carried out using KAPA2G™ Fast HotStart DNA Polymerase Polymerase. The sequences of primers used for the bisulfite sequencing analysis are presented in Additional file 1: Table S2. PCR products were run on 1.5% agarose gels and bands were excised using the TaKaRa MiniBEST Agarose Gel DNA Extraction Kit following the manufacturer’s instructions (TaKaRa). Purified bands were cloned using the pMD™19-T Vector Cloning Kit following the manufacturer’s instructions (TaKaRa). Colonies were selected and grown overnight in Luria-Bertani medium containing ampicillin (100 μg/ml) with shaking at 37 °C. Plasmid DNA was isolated using the TaKaRa MiniBEST Agarose Gel DNA Extraction Kit following the manufacturer’s instructions (TaKaRa). Plasmids were sequenced using the M13 universal reverse primer (BGI).

### Ectopic bone formation

In-vivo studies were performed with the approval of the Animal Experimentation Ethics Committee of The Chinese University of Hong Kong. After anesthesia, an incision was made on the dorsum and a subcutaneous pocket was created. 2.5 × 10^6^ ATSCs or BMSCs were seeded onto sterilized Skelite® resorbable Si-TCP bone graft substitute, and Si/TCP cubes with PBS served as the control group. The cells were then transplanted into the same mice. The wound was then closed in layers. At week 8, the scaffolds with cells were harvested for HE staining, as well as immohistochemical staining of osteocalcin (OCN). The osteoid matrix areas were measured using ImageJ software, and five microscopic fields were chosen from each sample and measured.

### Calvarial bone critical defect model

Six nude mice (6 weeks old, body weight 50 g) were used. All animals were placed under general anesthesia with a dosage of 0.2 ml/100 g body weight via intraperitoneal injection of a combination of ketamine, xylazine, and saline at a ratio of 3:2:3. The dorsal part of animal’s cranium was shaved and disinfected with iodine solution. The skin and underlying tissues including the periosteum were detached to expose the parietal bones on both sides. One piece of circular bone was removed in the middle region of the cranium using a hollow trephine bur with a 5-mm outer diameter. Continuous irrigation with sterile PBS was used to prevent overheating of the bone margins and to maintain moisture in the tissue. Any animal with evidence of meninges injury or continuous hemorrhaging was excluded. Then 50 μl of 2% hyaluronic acid hydrogel (5-mm-diameter cylinder) with 1 × 10^5^ human ATSCs or BMSCs was immediately implanted into the defect cavity. The periosteum and scalp were closed by suture. Animals were allowed to move following recovery from the anesthesia and were sacrificed by overdose of pentobarbital 6 weeks after surgery. The defect sites were removed, including sufficient parietal bone and soft connective tissues surrounding the defect areas.

### Micro-computed tomography imaging analysis

Micro-computed tomography (microCT) was used for quantitative evaluation of the bone formation. The samples were imaged using a high-resolution 70-kVp scan by microCT machine (VivaCT; Scanco Medical, Bassersdorf, Switzerland). The 3D reconstruction was performed using standardized segmentation parameters (sigma 0.8, threshold 160–1000), which were kept constant through the scan. Circular contour lines were drawn around the defect area (diameter = 5 mm) excluding the neighboring native bone. The 3D reconstructive images of samples were generated from 2D slices by machine built-in software. The bone volume within the selected circular defect was calculated using the quantitative 3D evaluation program included in the microCT software package.

### Histology and immunohistochemistry

Immunohistochemical staining was performed as described previously [23]. The samples were washed in PBS, fixed in 4% paraformaldehyde, decalcified, dehydrated, and embedded in paraffin. Sections were cut at a thickness of 5 μm and were stained with HE after deparaffination. Endogenous peroxidase activity was quenched with 3% hydrogen peroxide for 20 min at room temperature. Antigen retrieval was then performed with citrate buffer at 80 °C for 10 min for immunohistochemistry detection. Primary antibody against osteocalcin (1:100, sc-365797; Santa Cruz, CA, USA) was used. Donkey anti-goat IgG horseradish peroxidase (HRP)-conjugated secondary antibody was then added for 1 hour, followed by 3,3′-diaminobenzidine tetrahydrochloride (DAKO, Glostrup, Denmark) in the presence of H_2_O_2_ for signal detection of osteocalcin. Afterward, the sections were rinsed, counterstained in hematoxylin, dehydrated with graded ethanol and xylene, and mounted with *p*-xylene-bis-pyridinium bromide (DPX) permount (Sigma-Aldrich). Primary antibody was replaced with blocking solution in the negative controls. All incubation times and conditions were strictly controlled. The sections were examined under light microscopy (DMRXA2; Leica Microsystems Wetzlar GmbH, Germany).

### Data analysis

All experiments were performed at least three times. All data were expressed as the mean ± SD. The data were analyzed by independent two-tailed Student’s *t* test using SPSS (version16.0; Chicago, IL, USA). *p* < 0.05 was regarded as statistically significant.

## Results

### Characterize ATSCs and BMSCs with flow cytometry

**Fig. 1:**
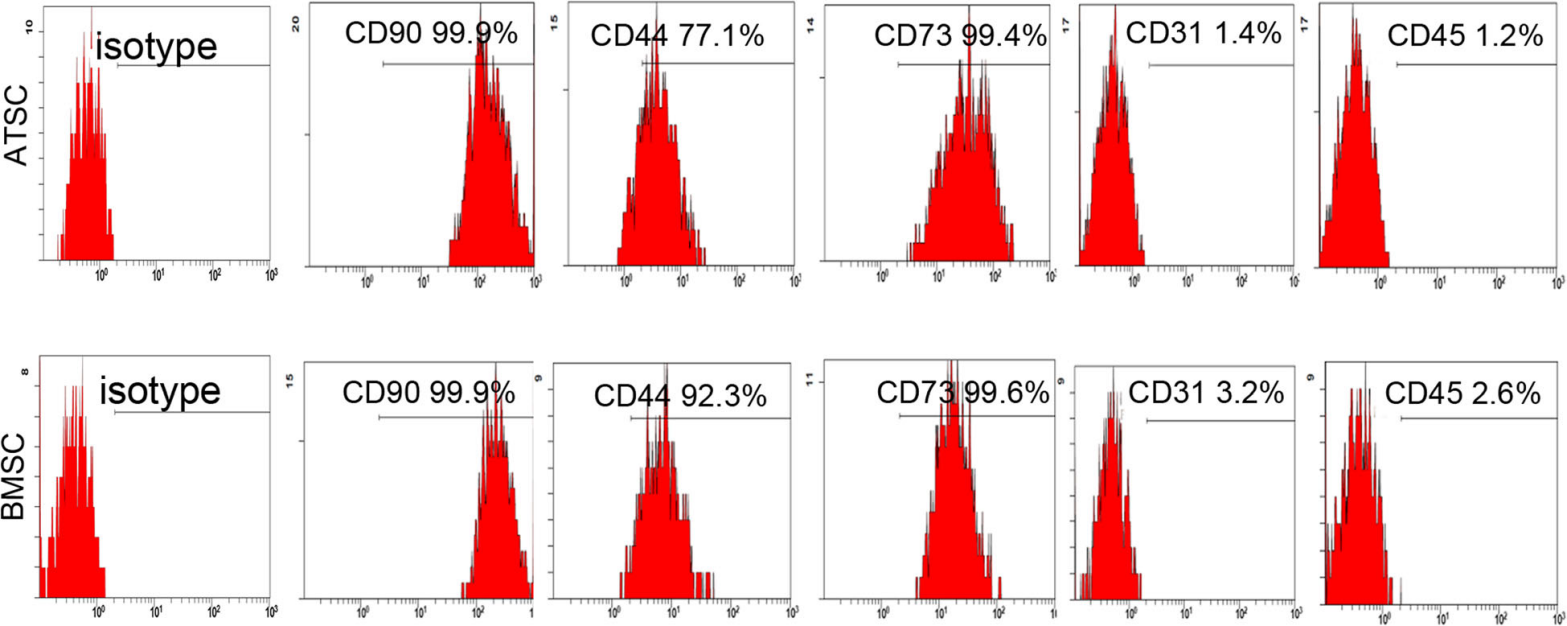
Characterization of cell surface markers of ATSCs and BMSCs. Cell surface markers of ATSCs and BMSCs (both at passage 3) were analyzed using flow cytometry. Antibodies against CD90, CD44, CD73, CD31, and CD45 were used to characterize ATSCs and BMSCs. ATSC: adipose tissue-derived MSC, BMSC: bone marrow-derived MSC, MSC: mesenchymal stem cell

The surface antigens of human ATSCs and BMSCs were detected by flow cytometry. The results showed that the cells were positive for CD90, CD44, and CD73 and negative for CD31 and CD45 (Fig. 1). The data showed that the cells expressed typical surface markers of MSCs and therefore were used for the experiments described in the following.

### Compare osteogenesis of ATSCs and BMSCs in vitro

**Fig. 2:**
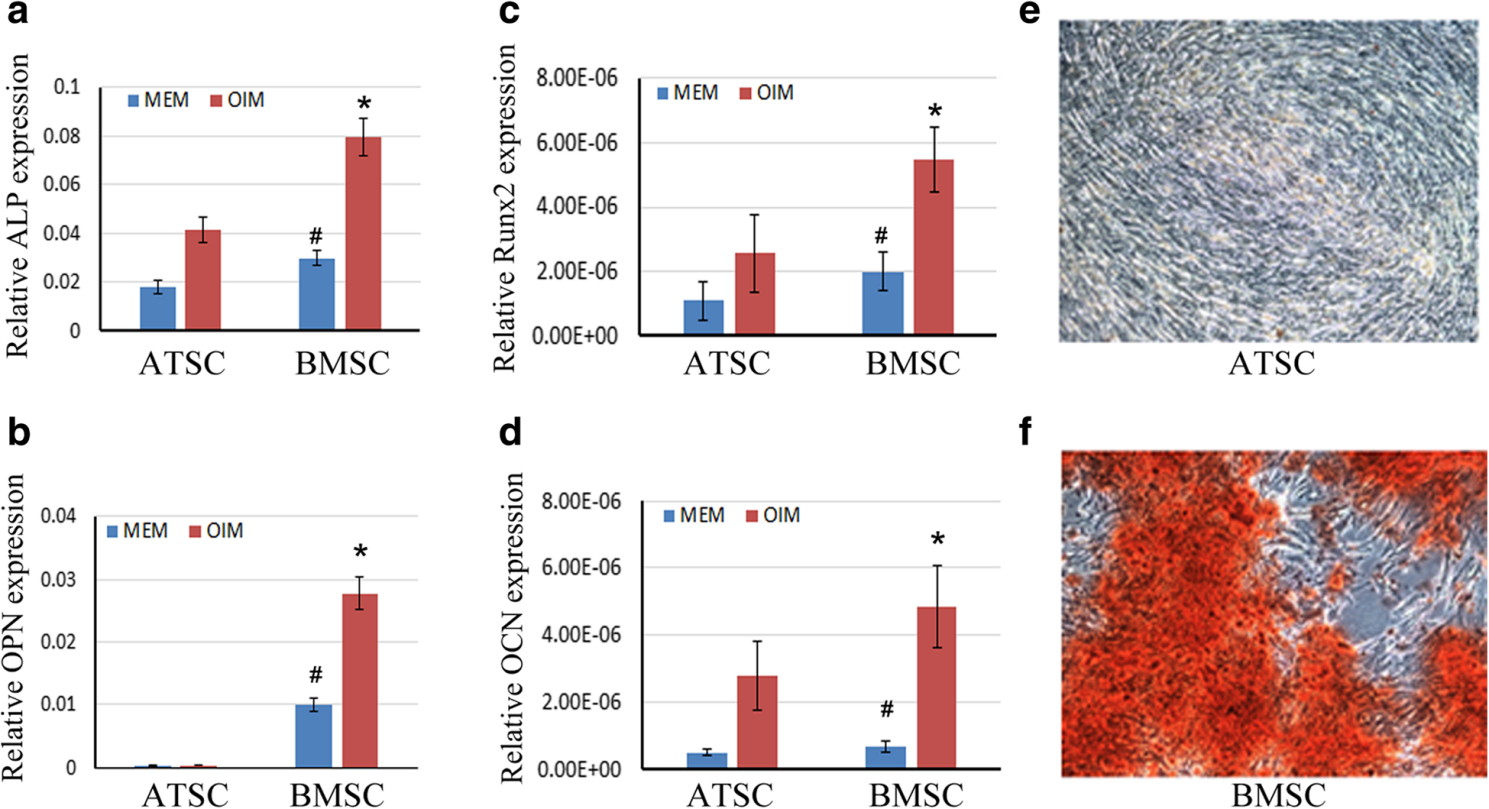
Difference in osteogenesis ability between ATSCs and BMSCs in vitro. **a**–**d** Total RNA extracted from ATSCs and BMSCs or cells subjected to OIM for 7 days. Relative expression levels of ALP, Runx2, OPN, and OCN checked by qRT-PCR. β-actin as an internal control. Data expressed as mean ± SD (*n* = 3). *p < 0.05, compared with MSCs in OIM; #*p* < 0.05, compared with MSCs in α-MEM. **e, f** Alizarin Red S staining of calcium deposits formed by ATSCs and BMSCs. ATSCs and BMSCs cultured in OIM for 14 days, then cells fixed and stained with Alizarin Red S. ALP: alkaline phosphatase, ATSC: adipose tissue-derived MSC, BMSC: bone marrow-derived MSC, MEM: minimum essential medium, MSC: mesenchymal stem cell, OCN: Osteocalcin, OIM: osteogenic induction medium, OPN: Osteopontin, Runx2: runt-related transcription factor 2

In order to compare the osteogenic differentiation potential capacities of ATSCs and BMSCs, MSCs were treated with OIM for several days and then the mRNA expression levels of genes related to osteogenesis were detected by quantitative real-time RT-PCR (qRT-PCR). As shown in Fig. 2a–d, the expression levels of alkaline phosphatase (ALP) and runt-related transcription factor 2 (Runx2), which are early markers for osteogenic commitment, were markedly increased in BMSCs compared with the ATSCs, as well as the late osteogenic markers Osteocalcin (OCN) and Osteopontin (OPN). To confirm the osteogenic commitment of BMSCs and ATSCs, Alizarin Red S staining was used to detect the formation of calcium deposit. The results showed that after 14 days of OIM induction, mineralization was seen in BMSCs upon osteogenic induction, while there were very few Alizarin Red S-positive calcium nodules formed in the ATSC group (Fig. 2e, f). These data indicated that BMSCs possessed higher potential for differentiation into osteoblasts compared to ATSCs.

### Compare adipogenesis of ATSCs and BMSCs in vitro

**Fig. 3:**
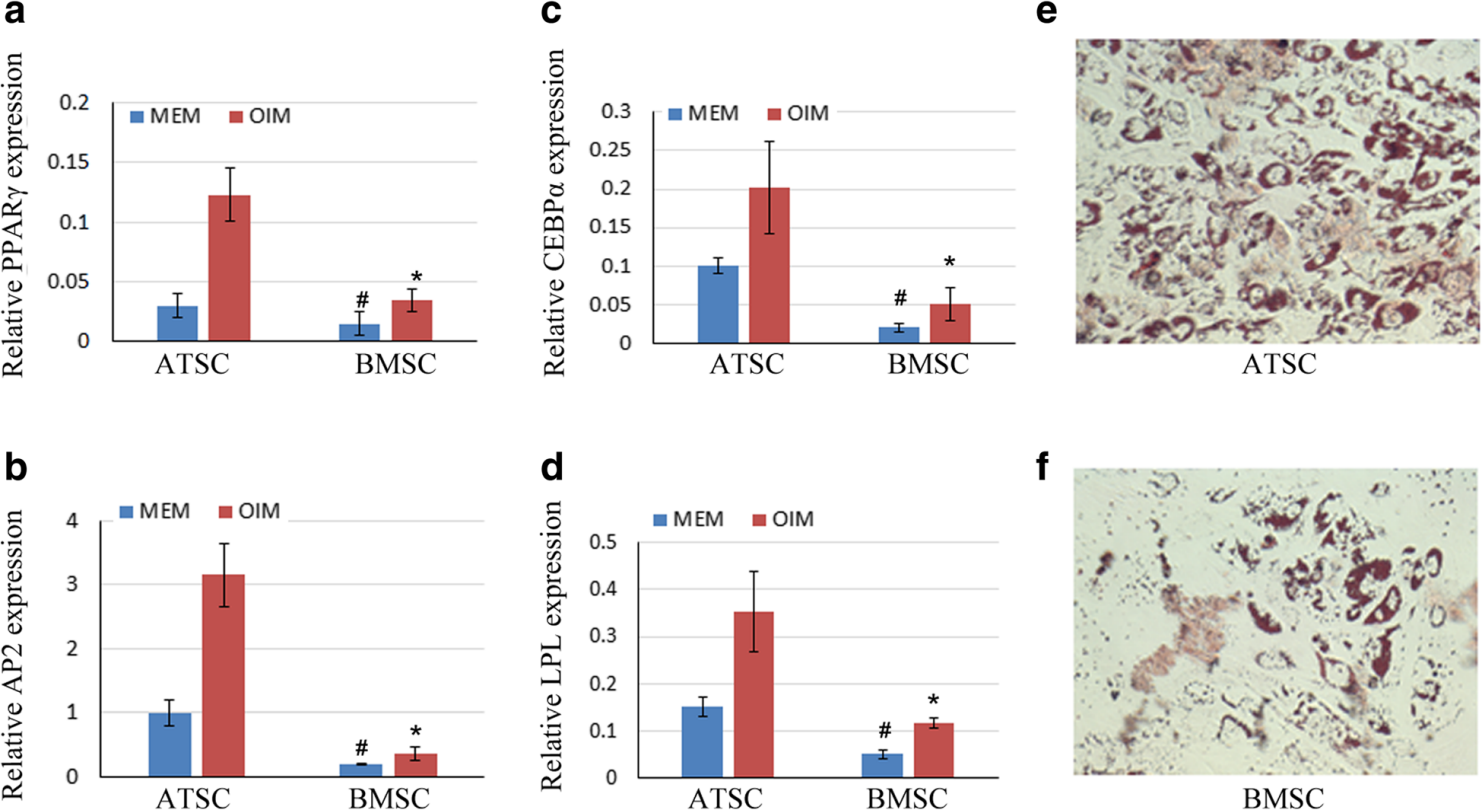
Difference of adipogenesis between ATSCs and BMSCs in vitro. **a**–**d** Total RNA extracted from ATSCs and BMSCs when cells were subjected to AIM for 7 days. Relative expression levels of PPARγ, CEBPα, AP2, and LPL checked by qRT-PCR. β-actin as an internal control. Data expressed as mean ± SD (*n* = 3). *p < 0.05, compared with MSCs in OIM; #*p* < 0.05, compared with MSCs in α-MEM. **e, f** ATSCs and BMSCs cultured in AIM for 21 days, then cells fixed and stained with Oil Red O. AP2 adipocyte protein 2, ATSC adipose tissue-derived MSC, CEBPα CCAAT/enhancer-binding protein alpha, BMSC bone marrow-derived MSC, LPL lipoprotein lipase, MEM minimum essential medium, MSC mesenchymal stem cell, OIM osteogenic induction medium, PPARγ peroxisome proliferator-activated receptor gamma

Next, we evaluated the adipogenic differentiation potential capacities of ATSCs and BMSCs. The cells were treated with AIM for several days, and then the mRNA expression levels of genes related to adipogenesis were detected by qRT-PCR. Our results showed that the expression levels of adipogenesis-related marker genes such as peroxisome proliferator-activated receptor gamma (PPARγ), CCAAT/enhancer-binding protein alpha (CEBPα), adipocyte protein 2 (AP2), and lipoprotein lipase (LPL) were significantly increased in ATSCs compared with the BMSCs (Fig. 3a–d). After 21 days of AIM induction, the cells were fixed for Oil Red O staining. The result showed that BMSCs had lower adipogenic differentiation potential as compared with ATSCs (Fig. 3e, f).

### DNA methylation analysis of main transcription factors

**Fig. 4:**
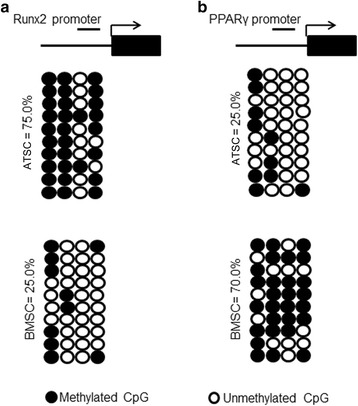
DNA methylation status of Runx2 and PPARγ in ATSCs and BMSCs. DNA methylation status of Runx2 (**a**) and PPARγ (**b**) promoters in ATSCs and BMSCs were examed using sodium bisulfite sequencing. Top panel indicates CpG dinucleotide position of the Runx2 and PPARγ promoter regions. Each PCR product was subcloned and subjected to nucleotide sequencing analysis. Ten representative sequenced clones depicted by filled (methylated) and open (unmethylated) circles for each CpG site. ATSC: adipose tissue-derived MSC, BMSC: bone marrow-derived MSC, MSC: mesenchymal stem cell, PPARγ: peroxisome proliferator-activated receptor gamma, Runx2: runt-related transcription factor 2

Because epigenetic regulation is an important factor to control MSC differentiation and the methylation status of DNA is the most common epigenetic modification of the genome in mammalian cells [24], we asked whether DNA methylation was involved in fate determination of ATSCs and BMSCs. Runx2 and PPARγ are the main master transcriptional factors controlling osteogenesis and adipogenesis, respectively. So, revealing the DNA methylation status of these two transcription factors may demonstrate their relationship with MSC fate determination. We calculated the percentage of methylated CpG loci (percent CpG methylation) in the total four CpG loci in Runx2 promoter and in four CpG loci in PPARγ promoter, respectively. We found that Runx2 promoter was hypermethylated whereas PPARγ promoter was hypomethylated in ATSCs (75% and 25% CpG methylation) (Fig. 4a, b). On the other hand, the methylation status of Runx2 was hypomethylated and PPARγ promoter was hypermethylated. These data suggest that DNA demethylation could be involved, at least partially, in the regulation of Runx2 and PPARγ in ATSCs and BMSCs; the source of MSCs is a direct factor influencing fate determination of MSCs.

### Compare chondrogenesis of ATSCs and BMSCs in vitro

**Fig. 5:**
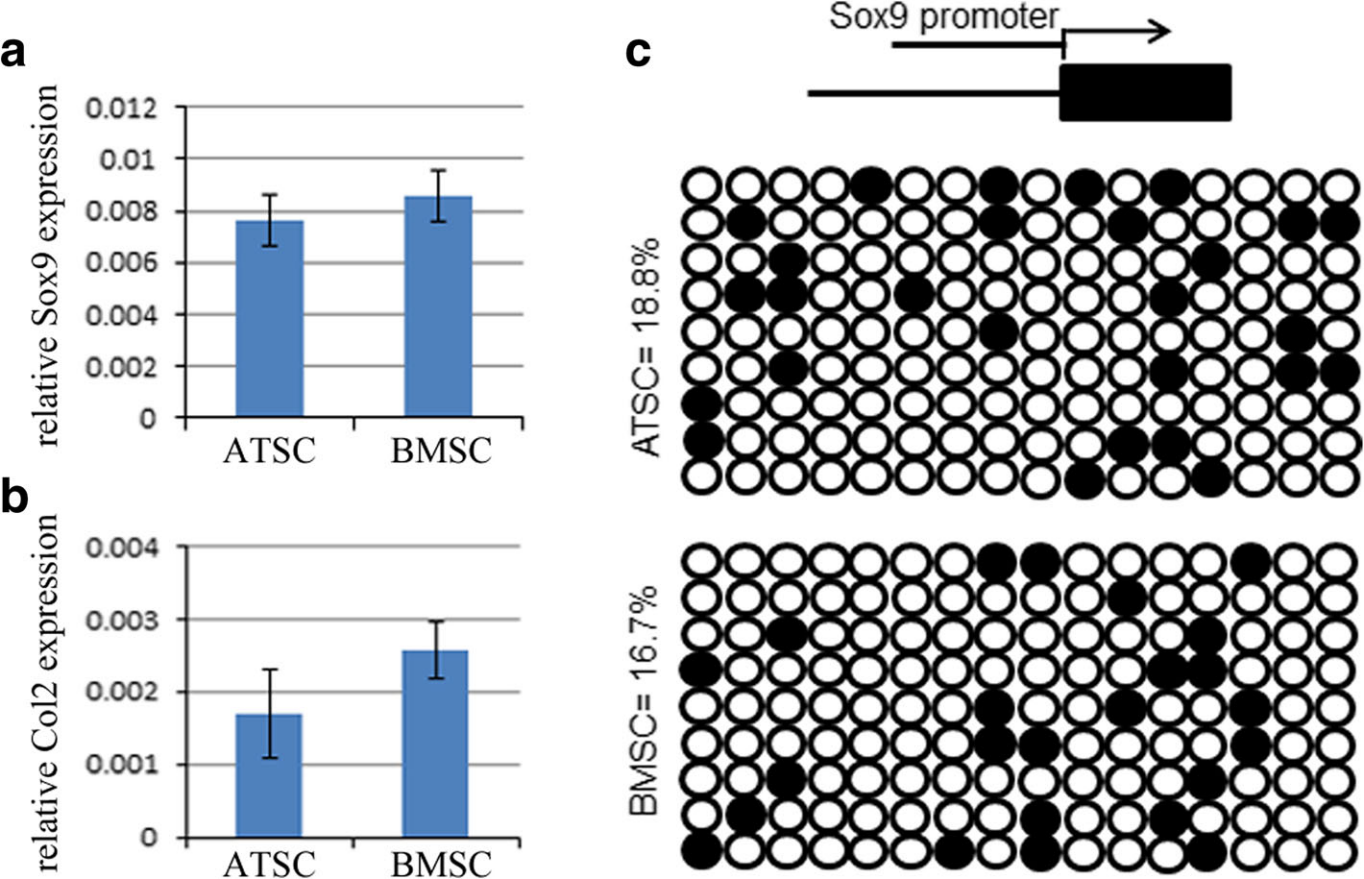
BMSCs showed slightly stronger chondrogenic differentiation potential. **a, b** Total RNA extracted from ATSCs and BMSCs when cells were subjected to CIM for 10 days. Relative expression levels of Sox9 and Col2 checked by qRT-PCR. β-actin as an internal control. Data expressed as mean ± SD. *p* < 0.05. **c** DNA methylation status of Sox9 promoter in ATSCs and BMSCs using sodium bisulfite sequencing. Top panel indicates CpG dinucleotide position of the Sox9 promoter region and numbers show positions of CpGs relative to the translation start site. Each PCR product was subcloned and subjected to nucleotide sequencing analysis. Ten representative sequenced clones depicted by filled (methylated) and open (unmethylated) circles for each CpG site. ATSC: adipose tissue-derived MSC, Col2: Collagen type II, BMSC: bone marrow-derived MSC, MSC: mesenchymal stem cell

Next, we wanted to know whether there is any difference in chondrogenesis ability between ATSCs and BMSCs. The cells were treated with CIM for 10 days, and then the expression levels of Sox9 and Collagen type II were evaluated by qRT-PCR. The results showed that both Sox9 and Collagen type II were slightly lower in ATSCs (Fig. 5a, b). Further bisulfite sequencing data showed that there was no significant difference in the methylation status of CpG sites in the promoter of Sox9 between ATSCs and BMSCs (Fig. 5c).

### Ectopic bone formation of ATSCs and BMSCs in vivo

**Fig. 6:**
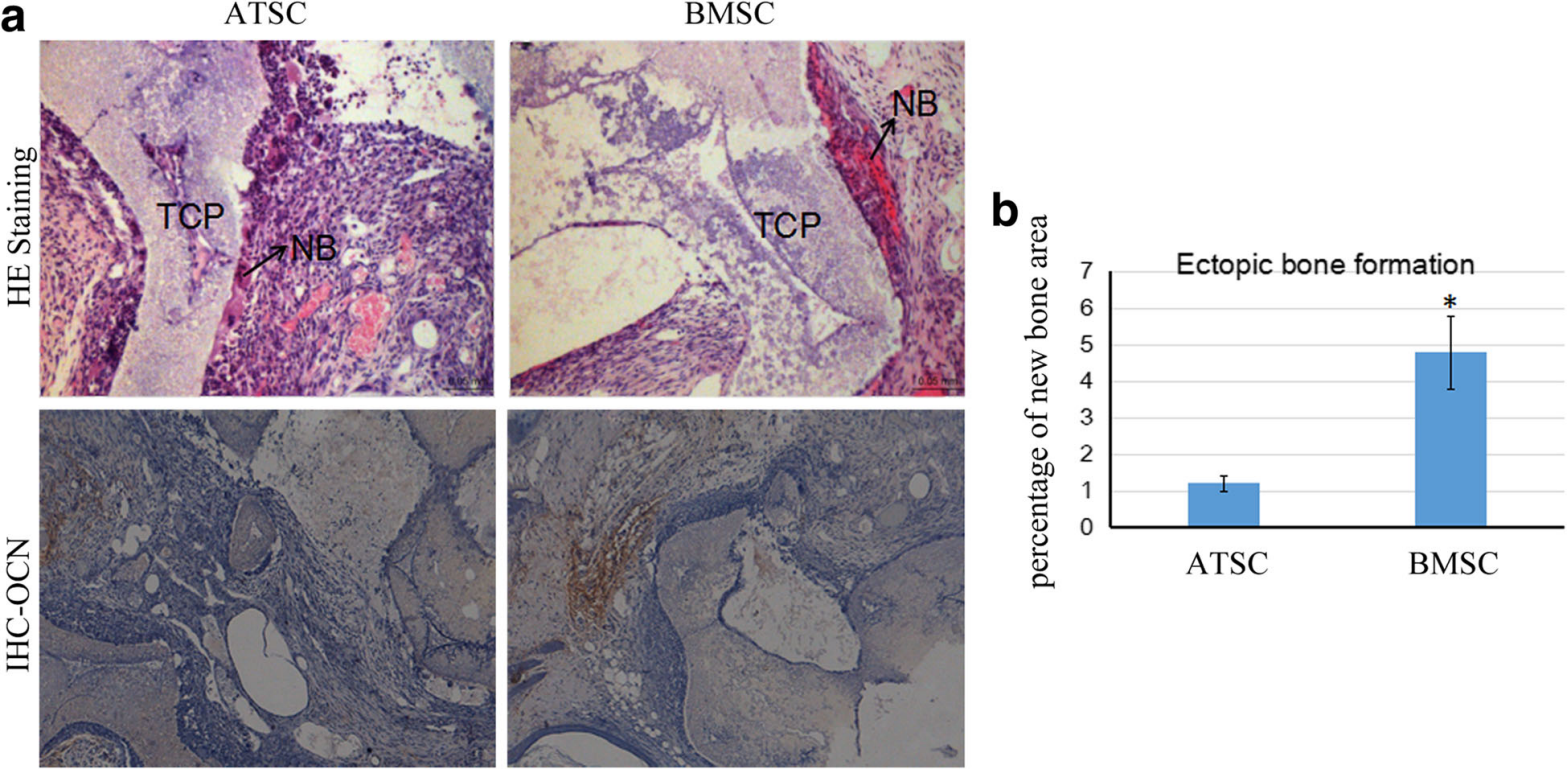
Ectopic bone formation of ATSCs and BMSCs in nude mice. **a**, ATSCs and BMSCs were loaded onto sterilized porous calcium phosphate restorable granules, then implanted subcutaneously into the dorsal surfaces of nude mice. Transplants were harvested 8 weeks later for histological examination. Sections stained with routine hematoxylin and eosin (HE) and immunohistochemical (IHC) staining with anti-OCN antibody. **b**, The bone formation area were measured and there were significantly more bone formation in the BMSC group, *p,<0.05. ATSC: adipose tissue-derived MSC, BMSC: bone marrow-derived MSC, MSC: mesenchymal stem cells, NB: new bone tissue, OCN: Osteocalcin

To further evaluate the advantages of BMSCs in osteogenic differentiation in vivo, BMSCs and ATSCs were loaded onto sterilized Skelite® resorbable Si-TCP bone graft substitutes respectively and implanted subcutaneously at the dorsal sides of nude mice. The transplants were harvested 8 weeks later and subjected to histological examination with HE staining or immunohistochemical analysis to detect the distribution of osteoid and the expression of OCN. Our results showed that transplantation of BMSCs with Si-TCP resulted in more bone-like tissue formation and less loose fibrous tissue and adipose tissue formation around the scaffold compared to the ATSCs with Si-TCP in nude mice. The formation of bone-like tissue was confirmed by the presence of osteocalcin (Fig. 6a, b). These results indicated that BMSCs were superior to ATSCs in ectopic bone formation in vivo.

### Bone regeneration in vivo using the calvarial defect model

**Fig. 7:**
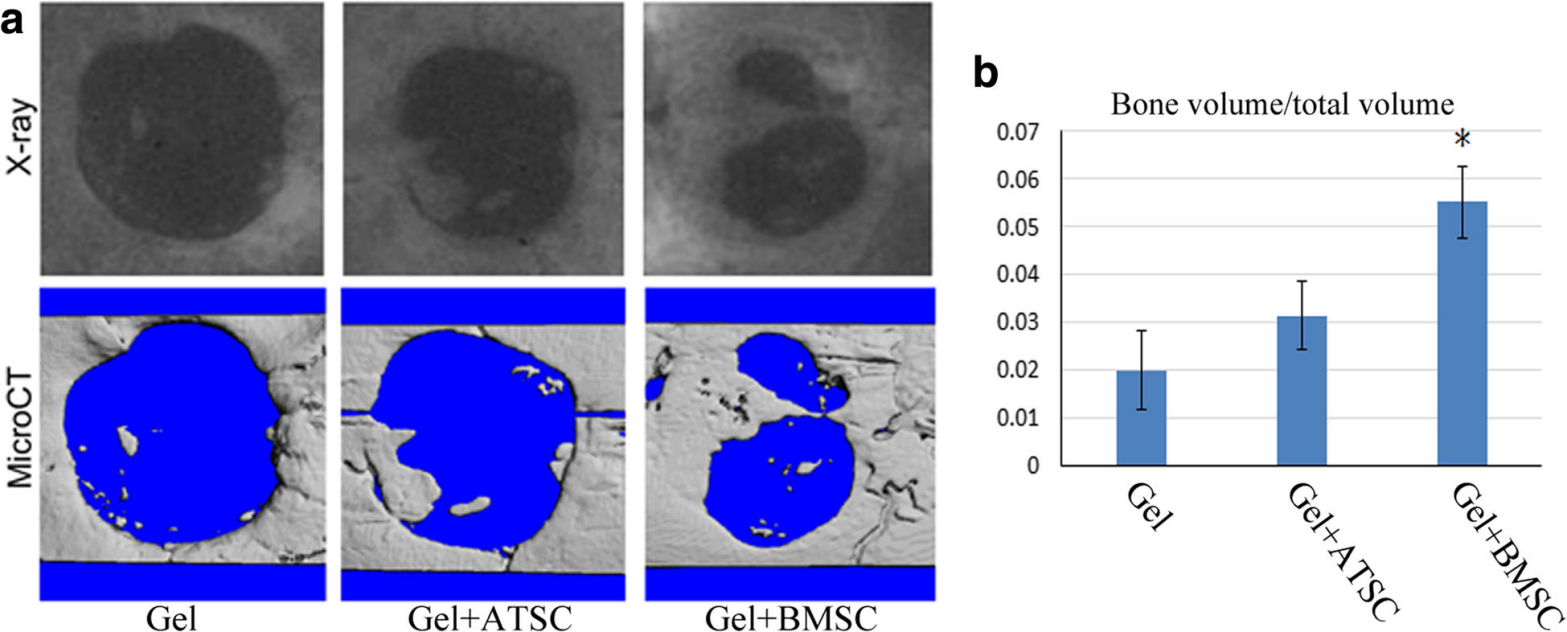
ATSCs and BMSCs enhanced calvarial bone repair in nude mice. **a** X-ray and microCT analysis 3D reconstruction of calvarial bone samples. **b** MicroCT analysis showed the new bone volume in the BMSC group was significantly increased (*) compared to that of the control group. ATSC adipose tissue-derived MSC, BMSC bone marrow-derived MSC, CT computed tomography, MSC mesenchymal stem cell

To compare the effect of ATSCs and BMSCs on bone repair, the nude mice calvarial bone critical defect model was used. Then 50 μl of 2% hyaluronic acid hydrogel (5-mm-diameter cylinder) with 1 × 10^5^ human ATSCs or BMSCs was immediately implanted into the defect cavity. Six weeks later, the samples were collected for X-ray and microCT reconstruction analysis. The result showed that more new bone tissue was observed in the BMSC group compared with the ATSC group (Fig. 7a, b). The ratio of bone volume/total volume in the BMSC group was significantly increased compared to that of the ATSC group (Fig. 7a, b).

## Discussion

In the present study, we compared the differentiation capacities of ATSCs and BMSCs, and demonstrated that BMSCs possessed stronger osteogenic but lower adipogenic differentiation potentials compared to ATSCs. There was no significant difference between their chondrogenic differentiation potential. Interestingly, our results provided important evidence that the DNA methylation status of the master transcription factors controlling MSC fate determination was responsible for the regulation of MSC differentiation capacities. The epigenetic memory obtained from either bone marrow or adipose tissue favored their differentiation along osteoblastic or adipocytic lineage. This is of particular interest, since the finding provided a potential explanation to elucidate the mechanism which was responsible for the regulation of MSC differentiation capacities by the origin they derived.

MSCs have been isolated from the umbilical cord, umbilical cord blood, bone marrow, adipose tissue, and many other adult tissues. They have been reported to share similar characteristics in vitro, such as plastic adherence, proliferation capacity, immunophenotype, and multilineage differentiation ability [25]. Lee et al. [26] reported that ATSCs were superior to BMSCs with respect to their maintenance of proliferating ability, but had similar morphology, phenotype, and microarray analysis of gene expression, and did not reveal differentially expressed osteogenic or adipogenic related genes between ATSCs and BMSCs. Our results have also provided evidence that ATSCs and BMSCs have similar morphology and cell surface markers, but their differentiation capacity was different. However, the intrinsic molecular mechanism supporting these observations has been lacking. We have partially addressed this question by exploring the methylation status of promoters of main transcription factors regulating trilineage differentiation potentials of MSCs.

DNA methylation is one of the most important epigenetic regulations which possesses the power to control the differentiation of or maintain the self-renewal of MSCs [17]. Accumulating evidence has demonstrated that changes in the methylation states of the CpG islands in the promoter regions or the first exon are inversely responsible for expression of the corresponding genes [27, 28]. The bivalent loci in MSCs are often reduced in DNA methylation and can be further methylated or activated, distinct from those in embryonic stem cells and differentiated cells [18]. A recent study demonstrated that the promoter regions of key genes in osteogenic differentiation such as BMP2 and ALP are epigenetically locked in MSCs to prevent their expression in nonosteogenic cells [29].

Sørensen et al. [30] reported that MSC differentiation did not affect lineage-specific promoter methylation states, arguing that these methylation patterns in differentiated cells are already established at the progenitor stage. But they did not compare the methylation status of the main transcription factors from different source-derived MSCs. We produced bisulfite PCR analysis to check the DNA methylation status of Runx2, PPARγ, and Sox9 from ATSCs and BMSCs. We demonstrated that BMSCs possessed stronger osteogenic and lower adipogenic differentiation potentials, which is completely different from ATSCs. The conclusion is that the differentiation potential of MSCs is highly influenced by their tissue of origin through epigenetic regulations such as DNA methylation of important transcription factors.

MSCs hold great promise for the treatment of a variety of difficult diseases such as myocardial infarction [31], neural diseases [32], and bone and cartilage defect [33, 34]. Bone marrow is the one of the major sources of MSCs, where they represent only approximately 0.001–0.01% of the nucleated cells, which is much less abundant than hematopoietic stem cells (HSCs). Adipose tissue is the main alternative source of MSCs and is more abundant and easier to isolate. Which type of MSCs is more suitable for clinical application? Some studies indicate that the clinical application potential of ATSCs is more effective or as effective as that of BMSCs, while others conclude that BMSCs are superior to ATSCs [11–13]. In this study, we isolated bone marrow and adipose-derived MSCs from people aged older than 60 years. ATSCs and BMSCs from the same donors were evaluated. The results showed that BMSCs and ATSCs exhibited different trilineage differentiation potentials, although they expressed similar cell surface makers and had a similar phenotype. The bisulfite sequencing data further provided a mechanism basis to make this conclusion, which explicated why MSCs from bone marrow were better for bone regeneration. The differential ability in trilineage differentiation is determined by the origin of MSCs. Checking DNA methylation of the main transcription factors governing MSC differentiation may be a predictive approach to distinguish different subpopulations of MSCs which may lead to better outcome for tissue regeneration. For example, MSCs with a lower DNA methylation rate in Runx2 promoter and a higher methylation rate in PPARγ promoter would result in better bone regeneration.

## Conclusion

Taken together, our results demonstrated that the methylation status of the main transcription factors controlling MSC fate influenced their expression, which resulted in the different differentiation capacities of ATSCs and BMSCs. This study has provided strong evidence that BMSCs are superior to ATSCs in terms of osteogenic differentiation rather than adipogenic differentiation, which is meaningful for the application of BMSCs in tissue engineering and regeneration of bone instead of ATSCs.

## Additional file

Additional file 1:
**Table S1.** Sequences of primers for real-time PCR analysis, **Table S2.** Sequences of primers for bisulfite sequencing analysis, and **Table S3.** Sequences of primers for CHIP-PCR analysis.

## Abbreviations

AIM: Adipogenic induction medium
ALP: Alkaline phosphatase
AP2: Adipocyte protein 2
ATSC: Adipose tissue-derived MSC
BMSC: Bone marrow-derived MSC
CEBPα: CCAAT/enhancer-binding protein alpha
CIM: Chondrogenic induction medium
HSC: Hematopoietic stem cell
LPL: Lipoprotein lipase
MSC: Mesenchymal stem cell
OCN: Osteocalcin
OIM: Osteogenic induction medium
OPN: Osteopontin
PBS: Phosphate-buffered saline
PPARγ: Peroxisome proliferator-activated receptor gamma
Runx2: Runt-related transcription factor 2
α-MEM: Minimum Essential Media alpha
