# Supplementary material for: Tissue source determines the differentiation potentials of mesenchymal stem cells: a comparative study of human mesenchymal stem cells from bone marrow and adipose tissue
Source: Stem Cell Res Ther. 2017 Dec 6;8:275. doi: 10.1186/s13287-017-0716-x (PMC5718061; doi:10.1186/s13287-017-0716-x)
Supplement: Additional file 1: — Table S1. Sequences of primers for real-time PCR analysis, Table S2. Sequences of primers for bisulfite sequencing analysis, and Table S3. Sequences of primers for CHIP-PCR analysis. [file 13287_2017_716_MOESM1_ESM.doc]

Additional file 1

**Table S**1. Sequences of primers for real time PCR analysis

| Gene Name | Forward primer sequence (5’ to 3’) | Reverse primer sequence (5’ to 3’) |
| --- | --- | --- |
| *OPN* | GTACCCTGATGCTACAGACG | TTCATAACTGTCCTTCCCAC |
| *OCN* | CACTCCTCGCCCTATTGGC | CCCTCCTGCTTGGACACAAAG |
| *Runx2* | ACTTCCTGTGCTCGGTGCT | GACGGTTATGGTCAAGGTGAA |
| *GAPDH*  *ALP*  *PPARγ*  *CEBPα*  *AP2*  *LPL*  *SOX9*  *COL2* | GGCATGGACTGTGGTCATGAG  CTCCCAGTCTCATCTCCT  GAAACTTCAAGAGTACCAAAGTGCAA  ACTTGGTGCGTCTAAGATGAGGG  AAAGAAGTAGGAGTGGGCTTTGC  AAAGAAGTAGGAGTGGGCTTTGC  GTACCCGCACTTGCACAAC  CCGAGGCAACGATGGTCAGC | TGCACCACCAACTGCTTAGC  AAGACCTCAACTCCCCTGAA  AGGCTTATTGTAGAGTCTGAGTCTTCTC  CATTGGAGCGGTGAGTTTGC  CCCCATTCACACTGATGATCAT  CCCCATTCACACTGATGATCAT  TCTCGCTCTCGTTCAGAAGTC  TGGGGCCTTGTTCACCTTTGA |

**Table S**2. Sequences of primers for bisulfite sequencing analysis

| Promoters | Forward primer sequence (5’ to 3’) | Reverse primer sequence (5’ to 3’) |
| --- | --- | --- |
| *Runx2* | AGAGTAAGGGGGAAAAGTTATAGTG | AAAAACACTCACTAACTCTATTAATCTC |
| *PPARγ*  *Sox9* | GAATGAATAGTTATTTAATGGAGATTAATT  GGTTGGAGAATGATTTGTTAGAGTT | CTCAAAACCTTTACCCTTTTTAACA  CAATAAAAAACAAAACTAAATCCCC |

**Table S**3. Sequences of primers for CHIP-PCR analysis

| Promoters | Forward primer sequence (5’ to 3’) | Reverse primer sequence (5’ to 3’) |
| --- | --- | --- |
| *Nanog-1*  *Nanog-2* | GCTTGAGGGGGAGGAGTAAG  GCTTGAGGGGGAGGAGTAAG | GCTTGAGGGGGAGGAGTAAG  GCTTGAGGGGGAGGAGTAAG |
| *Oct4* | GTGAGTCGTCCTTCCACCAG | GAGAAGGCGAAGTCTGAAGC |
